# Allergenic Asteraceae in air particulate matter: quantitative DNA analysis of mugwort and ragweed

**DOI:** 10.1007/s10453-017-9485-3

**Published:** 2017-06-06

**Authors:** I. Müller-Germann, D. A. Pickersgill, H. Paulsen, B. Alberternst, U. Pöschl, J. Fröhlich-Nowoisky, V. R. Després

**Affiliations:** 1 Biogeochemistry and Multiphase Chemistry Departments, Max Planck Institute for Chemistry, Hahn-Meitner-Weg 1, 55128 Mainz, Germany; 2 Geosciences, Johannes Gutenberg University, Joh.-Joachim-Becher-Weg 21, 55128 Mainz, Germany; 3 Molecular Physiology, Johannes Gutenberg University, Joh.-von-Müller-Weg 6, 55099 Mainz, Germany; 4 Working Group Biodiversity and Landscape Ecology, Hinter’m alten Ort 9, 61169 Friedberg, Germany

**Keywords:** Bioaerosol, DNA, Real-time PCR, *Ambrosia artemisiifolia*, *Artemisia vulgaris*

## Abstract

Mugwort (*Artemisia vulgaris*) and ragweed (*Ambrosia artemisiifolia*) are highly allergenic Asteraceae. They often cause pollen allergies in late summer and fall. While mugwort is native to Europe, ragweed reached Europe as a neophyte from North America about 150 years ago and continued spreading ever since. To understand possible relationships between the spread of ragweed, its abundance in air, and to judge possible health risks for the public, we quantified ragweed DNA in inhalable fine as well as in coarse air particulate matter. Mugwort was chosen for comparison, as it is closely related to ragweed and grows in similar, though mainly not identical, habitats but is native to Germany. The DNA quantification was performed on atmospheric aerosol samples collected over a period of 5 years in central Europe. The DNA concentrations were highest during the characteristic pollination periods but varied greatly between different years. In the inhalable fine particle fraction, ragweed exceeds the mugwort DNA concentration fivefold, while the coarse particle fraction, bearing intact pollen grains, contains more mugwort than ragweed DNA. The higher allergenic potential of ragweed might be linked to the humidity or long-range transport-induced bursting of ragweed pollen into smaller allergenic particles, which may reach the lower airways and cause more intense allergic reactions. Airborne ragweed DNA was detected also outside the local pollination periods, which can be explained by atmospheric long-range transport. Back-trajectory analyses indicate that the air masses containing ragweed DNA during winter had originated in regions with milder climate and large ragweed populations (Southern France, Carpathian Basin).

**Electronic supplementary material:**

The online version of this article (doi:10.1007/s10453-017-9485-3) contains supplementary material, which is available to authorized users.

## Introduction

Mugwort (*Artemisia vulgaris*) and common ragweed (*Ambrosia artemisiifolia*) are highly allergenic weeds belonging to the Asteraceae family. In general, 10–14% of the already atopic individuals in Europe suffer from mugwort-caused pollinosis (Spieksma et al. 1980; Table ESM1). For ragweed, the numbers are even higher: Up to 50% of the atopic individuals in the North American and Canadian population are additionally allergic to ragweed pollen (Wopfner et al. 2005). Within Europe, the prevalence of ragweed sensitization among atopic individuals varies widely over a range of ~2% in Finland, ~15% in the Netherlands and Germany, and up to ~50% in Hungary (Burbach et al. 2009).

Mugwort is native to Europe and parts of Asia and grows in the temperate and humid zones of the northern hemisphere and the Mediterranean Basin (Barney and Di Tommaso 2003; Gadermaier et al. 2004; Wopfner et al. 2005). In Germany, mugwort is a widespread plant, which often grows at ruderal sites like footpaths, field edges, or at dumping grounds (Sebald et al. 1996) but avoids heavily disturbed sites. The surroundings of the sampling location in this study are no exception, with mugwort belonging to the very abundant plants of the region. The main pollination season in Central Europe is generally from July to August, although some pollen can already be observed as early as June and as late as September (D’Amato and Spieksma 1991; Grewling et al. 2012). The threshold which is sufficient to cause first allergic symptoms against mugwort has been reported to range from daily averages of 4–30 pollen per m^3^ of air, depending on the area (De Weger et al. 2013 and references therein).

In contrast to native mugwort, common ragweed originates from the North American plains and Canada and has colonized several countries of Southern, Central, and Eastern Europe since the second half of the nineteenth century (Dahl et al. 1999; Mandrioli et al. 1998). Currently, common ragweed is quite rare in most parts of Germany, except for some regions such as the area around the city of Cottbus where the species has become abundant (Alberternst et al. 2006; Brandes and Nitsche 2007; Buters et al. 2015; Lemke 2013; Nawrath and Alberternst 2010). As for the region surrounding our sampling site, reliable data on the abundancy of ragweed are hard to find. From our own experience, ragweed can be found growing in the region. According to Cunze et al. (2013), the habitat suitability of common ragweed under current climatic conditions in our study area is quite high and Buters et al. (2015) shows that all counties to north and east, in southern Hesse, reported ragweed populations of >100 individual plants, between 2000 and 2010 and furthermore, they show that in 2014 our sampling site lay in the region they classify as having an extensive ragweed population.

The pollination of ragweed plants in Central Europe starts in late summer. The first airborne pollen can be detected in July and has their highest concentrations in August and September, and pollen release can last until the first onset of frost (Alberternst et al. 2009; Dahl et al. 1999; Gadermaier et al. 2004) as the plant dies at temperatures under −5 °C (Dahl et al. 1999).

The increasing problem of sensitization to ragweed pollen in Europe has stimulated many studies on ragweed (Alberternst et al. 2006, 2009; Asero 2007; Brandes and Nitsche 2006; Burbach et al. 2009; Buttenschøn et al. 2009; Dahl et al. 1999; Fumanal et al. 2007; Jäger 2000; Kaczinczi et al. 2008; Kaminski et al. 2010; Kasprzyk et al. 2011; Makra et al. 2004; Mandrioli et al. 1998; Otto et al. 2008; Peternel et al. 2005, 2008; Piotrowska and Weryszko-Chmielewska 2006; Poppendieck von 2007; Smith et al. 2008; Stach et al. 2007; Ziska et al. 2007). In countries with extensive ragweed populations leading to an allergological relevant pollen charge in the air, the rate of sensitization is often high, e.g., up to ~50% in Hungary where large ragweed stocks are located (Burbach et al. 2009). In the north of Italy, for example, ragweed pollen allergy has become the second highest of all allergies in the past few decades, with an increase from no documented cases in the years before 1997 up to ~20% in 2007 (Asero 2007). Worldwide the prevalence for ragweed allergy has doubled in 20 years from 15% to 30% (Arbes et al. 2005). Studies, which elucidate the abundance, dispersion, and possible dependence of ragweed pollen distribution and load in the air on meteorological factors, are therefore needed. With the help of such studies, we can better understand possible future health impacts as well as more precisely predict the influence of climate change.

For the quantification of DNA contained in airborne plant material, this study concentrates on a molecular approach. Pollen determination under the light microscope is often not precise enough to go down to the genus or even species level (De Weger et al. 2013). More importantly, fragments of pollen and other plant material which also cause allergic symptoms cannot be identified with a microscopic approach (Otto et al. 2008; Wright 1963), and this is one of the main benefits of this studies approach as insight is gained into concentrations found in the fine particulate matter fraction, which can penetrate deep into the respiratory tract.

The aim of this study was to quantify and compare the concentrations of mugwort and ragweed DNA, which can originate from either pollen or plant tissue, over a 5-year period in Mainz, Germany, from both the coarse and fine particulate size fractions. Air filter samples from the plant-specific flowering seasons and from the non-flowering seasons in spring and winter were analyzed with ragweed- and mugwort-specific quantitative real-time PCR (qPCR) as already established in an exploratory study for birch DNA in Müller-Germann et al. (2015). In addition, qPCR results were statistically tested for correlation to meteorological parameters and wind back trajectories were interpreted. Furthermore, while the sensitization rate of the population seems to be continuously increasing, this study investigates whether this phenomenon may correspond to a simultaneous increase in aerosolized allergenic material over time. The cross-reactivity of mugwort and ragweed might be an important factor for sensitive individuals and thus underlines the need for a comparative study dealing with both allergenic weeds.

## Materials and methods

All the applied laboratory methods are described in more detail in Müller-Germann et al. (2015). Here, we present a more brief overview and focus on the methodological and analytical differences to Müller-Germann et al. (2015).

### Aerosol sampling

The aerosol sampling procedure has been described in detail in Fröhlich-Nowoisky et al. (2009) and is summarized as follows: Aerosol samples were collected on glass fiber filters (Pall Corporation, Type A/A, 102 mm diameter; sterilized at 500 °C for 12 h) over a 5-year period in Mainz, Germany (130 m above sea level (a.s.l.), March 2006–December 2010). The sampling station was located about 20 m above ground level, on the campus of the University of Mainz (49.99°N, 8.23°E). For the sampling, a high-volume dichotomous sampler (self-built as described in Solomon et al. (1983)) was used to separate and collect coarse and fine aerosol particles on a pair of glass fiber filters with a nominal cutoff at 3 µm. The sampler was operated with a rotary vane pump (Becker VT 4.25) at a total flow rate of ~300 L min^−1^. The sampled air masses represent a mix of urban and rural continental boundary layer air in central Europe. The sampling period was generally ~7 days, corresponding to a sampled air volume of ~3000 m^3^. A few samples were collected over shorter periods (1–5 days, ~400–2000 m^3^ air). Loaded filters were packed in aluminum foil (pre-baked at 500 °C) and stored at −80 °C until DNA extraction.

As listed in Table 1, for ragweed 67 filter pairs, comprised of a coarse (labeled a) and a fine (labeled b) particle filter, were analyzed. For mugwort, 89 particle filter pairs were analyzed. For both plants, filter samples from their pollination seasons (July–October) and their non-flowering seasons in spring (May) and winter (December) were chosen.

**Table 1:** Overview of mugwort and ragweed DNA quantification success in air (positive samples/analyzed samples)

Sampling period	Particle size fraction	Mugwort (*Artemisia vulgaris)*	Ragweed (*Ambrosia artemisiifolia*)
2006	Coarse	16/16	12/12
	Fine	10/16	12/12
2007	Coarse	16/16	12/12
	Fine	8/16	12/12
2008	Coarse	16/16	12/12
	Fine	6/16	12/12
2009	Coarse	14/19	14/14
	Fine	7/19	14/14
2010	Coarse	20/22	17/17
	Fine	12/22	17/17
2006–2010	Coarse	82/89	67/67
	Fine	43/89	67/67

### Primer design, testing and DNA extraction

The highly variable internal transcribed spacer (ITS) region of the multi-copy ribosomal DNA (rDNA) was chosen for analysis. Both forward primers are located within the ITS 1 region, whereas both reverse primers are located in the ITS 2 region (Figure ESM1 and Table 2). Primers were designed using Primer 3.0 with the forward primers in the ITS 1 region and the reverse primers in the ITS 2 regions (Rozen and Skaletsky 2003). They were tested using pDraw 32 (http://www.acaclone.com/) and through database comparison. The sensitivity was furthermore verified by PCR on dilution series down to ten DNA copies.

**Table 2:** Used primer pairs with their specific annealing temperatures

Primer	Sequence 5′-3′	Tm (°C)
Primer pair *Ambrosia artemisiifolia*		
*Ambrosia artemisiifolia* 62 for	CGG GGA TCG AAG CTT ATG T	55
*Ambrosia artemisiifolia* 640rev	GAA GCA TCA TCG CAA GAC AA	55
Primer pair *Artemisia vulgaris*		
*Artemisia vulgaris* 115 for	CTT TTG GAC CTC TTG TGA ATG CG	62
*Artemisia vulgaris* 460 rev	ATG TTC CCT TTG CGG AGA AAT	62

The location of the primer is visualized in Figure ESM1, Tm = Annealing temperature

DNA was extracted with a commercial soil DNA extraction kit (LysingMatrixE, Fast DNA Spin Kit for Soil, MP Biomedicals) with slight alterations to the supplier’s instructions (See Müller-Germann et al. 2015). For testing purposes, DNA was initially extracted from 50 mg of ragweed (*Ambrosia artemisiifolia*) and mugwort (*Artemisia vulgaris*) leaf material. For the qPCR measurements, DNA was extracted from air filter aliquots (1/8).

For verification, the test DNA extracts were amplified and cloned using the exact methods described in Müller-Germann et al. 2015. The products were sequenced at the DNA Core Facility of the Max Planck Genome Centre in Cologne using a ABI Prism Sequencer (Applied Biosystems, Darmstadt). The obtained sequences were submitted to the NCBI database and received the following accession numbers: KM487595-KM487598 (*Ambrosia artemisiifolia*) and KM487604-KM487611 (*Artemisia sp*.).

Quantitative real-time PCR (qPCR) was conducted to measure the amount of mugwort and ragweed DNA sequences. The Real-Time PCR MiniOpticon™ System for Real-Time PCR Detection (Biorad) was used for measurements using the Opticon Monitor™ Software (Version 3.1). Experimental setup and programming of the qPCR runs followed the supplier’s instructions using the iScript TM One-Step RT-PCR Kit (Biorad) with the primer-specific annealing temperatures (Table 2).

### Standard curves and preparation

The absolute quantification used in this study is based upon comparative measurements with a well-defined standard to determine the absolute amount of the target sequence. To prepare the standard dilutions, PCR products were cloned into *E.coli* Top 10 cells as described above. After 12 h of incubation from the grown colonies on the one hand, colony PCRs were performed and sequenced to validate the correct insertion and species specificity of the PCR product. On the other hand, these colonies were used to inoculate liquid media and grow pre-cultures. The selected colonies were transferred in 2 ml LB broth with 2 μl ampicillin (100 mg ml^−1^) and incubated for 15 h at 300 rpm and 37 °C. Subsequently, PCRs of the pre-cultures were performed, to check whether the vector contained the correct plant DNA fragment using the following restriction fragment length polymorphism (2 µl PCR product 5 units *Taq*I (Fermentas)). After gel electrophoresis restriction, fragment patterns were compared to theoretical restriction fragments calculated by pDraw 32 (http://acaclone.com/).

After verification, 100 μl of the pre-culture was transferred to 45 ml liquid LB medium containing 45 μl ampicillin (100 mg ml^−1^), which was incubated 15 h at 300 rpm and 37 °C. For the *E.coli* plasmid preparation of DNA, the GenElute™ Plasmid Miniprep Kit (Sigma-Aldrich) was used following the supplier’s instructions.

The concentration of the plasmid DNA was measured with Bio-Rad SmartSpec 3000 UV/Vis spectrophotometer using distilled water (dH_2_O) as a control. DNA extracts were diluted 1:10, and measurements were repeated at least four times for each plasmid preparation. These plasmid preparations were photometrically analyzed, and the ratio of A260/A280 was determined. For each plant, the plasmid preparation with the best ratio between 1.8 and 2.0 was chosen and used for the actual standard dilutions.

To calculate the plasmid copy number (CopyNo_Plasmid_) per µl, we used Eq. 1 using the measured plasmid preparation concentration (*C*_Plasmid_, g µl^−1^) as well as the known length (*L*_Plasmid_) and weight (*W*_Plasmid_, gmol^−1^) of an insert-containing plasmid (Lee et al. (2006) and Whelan et al. (2003)). See Table 3 for symbol explanation:

1 $${\text{CopyNo}}_{\text{Plasmid}} (\upmu{\text{l}}^{ - 1} ) = \frac{{N_A({\text{mol}}^{ - 1} ) \times C_{\text{Plasmid}}({\text{g }}\upmu{\text{l}}^{ - 1} )}}{{L_{\text{Plasmid}} \times W_{\text{Plasmid}} ( {\text{g mol}}^{ - 1} )}} = \frac{{ 6. 0 2\times 1 0^{ 2 3} ( {\text{mol}}^{ - 1} )\times C_{\text{Plasmid}} ({\text{g }}\upmu{\text{l}}^{ - 1} )}}{{L_{\text{Plasmid}} \times 6 6 0 {\text{ (g mol}}^{ - 1} )}}$$

**Table 3:** Equation parameters

Parameter	Quantity
CopyNo_Plasmid_	Copy number of the standard plasmid per µl extract
*C*_Plasmid_	Concentration of the plasmid (including PCR product (g µl^−1^)
*C*_t_	DNA concentration total (number of copies per cubic meter of air; m^−3^)
*C*_c_	DNA concentration coarse (number of copies per cubic meter of air; m^−3^)
*C*_f_	DNA concentration fine (number of copies per cubic meter of air; m^−3^)
*l*_Plasmid_	Length of plasmid DNA and included PCR product in base pairs (bp)
*m*_Plasmid_	Weight of plasmid and included PCR product in g mol^−1^
*N*_A_	Avogadro constant (mol^−1^)
*N*_t_	Number of DNA copies (cp), total
*N*_c_	Number of DNA copies (cp), coarse
*N*_f_	Number of DNA copies (cp), fine
*V*_c_	Sampled air for coarse flow (m^3^)
*V*_f_	Sampled air for fine flow (m^3^)
*V*_t_	Total air flow (m^3^)

For the standard curve, each preparation was then diluted in eight steps from 10^8^ to 10^1^ copies μl^−1^.

In each qPCR run, the standard was measured three times and each sample two times. The derived copy number of the PCR product was normalized by the sampled air volume. Therefore, the discussed copy numbers in this study refer to the copies per m^3^ of sampled air.

### Data analysis

The quality of a qPCR can be assessed by the PCR efficiency (E), which indicates how much template was amplified per cycle (Bustin et al. 2009; Pfaffl 2004). Quantitative PCR results with PCR efficiencies less than 80% were not used for further analysis. For each double replicate, the average initial quantity of the template (DNA copies: N_x_) and the number of DNA copies per m^3^ of sampled air (C_x_) were calculated: for coarse *V*_*c*_ and *C*_*c*_, for fine *V*_*f*_ and *C*_*f*_, and for the total particle size *V*_*tot*_ and *C*_*tot*_ (see Table 3 for equation parameter explanation, and calculations are given in Eqs. 2 and 3).

2 $$C_{\text{c}} = \left( {N_{\text{c}} - C_{\text{f}} \cdot V_{\text{c}}} \right){\raise0.7ex\hbox{$1$} \!\mathord{\left/ {\vphantom {1 {V{\text{tot}}}}}\right.\kern-0pt} \!\lower0.7ex\hbox{${V_{\text{tot}}}$}} = \left( {N_{\text{c}} - N_{\text{f}} \cdot {\raise0.7ex\hbox{${V_{\text{c}}}$} \!\mathord{\left/ {\vphantom {{V{\text{c}}} {V{\text{f}}}}}\right.\kern-0pt} \!\lower0.7ex\hbox{${V_{\text{f}}}$}}} \right) \cdot {\raise0.7ex\hbox{$1$} \!\mathord{\left/ {\vphantom {1 {V{\text{tot}}}}}\right.\kern-0pt} \!\lower0.7ex\hbox{${V{_\text{tot}}}$}}$$

3 $$C_\text{f} = \left( {N_\text{f} + N_\text{f} \cdot {\raise0.7ex\hbox{${V_\text{c}}$} \!\mathord{\left/ {\vphantom {{Vc} {Vf}}}\right.\kern-0pt} \!\lower0.7ex\hbox{${V_\text{f}}$}}} \right) \cdot {\raise0.7ex\hbox{$1$} \!\mathord{\left/ {\vphantom {1 {V{\text{tot}}}}}\right.\kern-0pt} \!\lower0.7ex\hbox{${V_{\text{tot}}}$}}$$

The DNA copies for coarse and fine particle filters were calculated separately, as 10% of the fine particles are sampled on the coarse particle filters due to the air flow of the virtual impactor, which is corrected in Eq. 2. However, the fine particle samples are essentially free from coarse particle contamination (Solomon et al. 1983). For further details, see also Müller-Germann et al. 2015.

### Quality control

To monitor contaminations during amplification and DNA extraction, in each qPCR run one negative control was included and extraction blanks were extracted along with sampled filters and analyzed in the qPCR. In Table ESM2, all analyzed blank samples are listed. To detect possible contaminations from the sampler and sample handling, blank samples were taken at regular intervals (~4 weeks). The blank filters were treated identically to the regular filters, but the pump was either not turned on at all (“mounting blanks”) or only for 5 s (“start-up blank”) as described in Fröhlich-Nowoisky et al. (2009). For both, mugwort and ragweed, 34 mounting and start-up blanks, respectively, were analyzed. During each extraction process, at least one blank filter was extracted in parallel and amplified in the qPCR run. For each qPCR run, a negative control was included to ensure a contamination-free amplification process and setup. For mugwort and ragweed, in total 20 extraction blanks were tested each.

No extraction blank, qPCR negative control, or sampling blank contained any DNA for ragweed, whereas for mugwort on one mounting and one start-up blank mugwort DNA was detected, however, both times only in one of the two replicates. Both blank samples were from the plant-specific main pollen season, which makes it more likely that minor amounts of mugwort may have settled on the filter while mounting it in the filter sampler (MZ 49a from 2006-08-02 and MZ 201a from 2008-08-07).

### Meteorological data

For the correlation analysis, the meteorological data for temperature, relative humidity, precipitation, and wind speed were provided by the Landesamt für Umwelt, Wasserwirtschaft und Gewerbeaufsicht Rhineland-Palatinate Zentrale Immessionsmessnetz (ZIMEN), which was gathered at their station in Mainz-Mombach, Germany. The dataset consists of half-hour values for all the observed sampling periods.

### Correlation analysis

Using the software environment for statistical computing and graphics, R (R Development Core Team 2011), individual sampling period averages, standard deviations, maximum and minimum values were calculated for temperature (°C), relative humidity (%), and wind speed (m s^−1^). For the precipitation, the sum of precipitation (mm), the duration of precipitation (h, with a half-hour resolution), and the average precipitation strength (mm h^−1^) were assessed for each sampling period. Single factor linear regression analysis was performed between meteorological factors and the corresponding copies per m^3^ for mugwort and ragweed [total suspended particles (TSP), coarse, and fine particle samples] for (a) the entire dataset, (b) for the entire dataset disregarding the values after each annual maximum copies per m^3^, (c) for each year individually, and (d) for the individual years up to the maximum copies per m^3^.

### Back-trajectory calculation

Back trajectories were calculated with Hysplit 4 (Draxler and Rolph 2003) for all of the sampling periods analyzed in this study using the Global Data Assimilation System (GDAS) meteorological datasets from the National Centers for Environmental Prediction (NCEP). The gridded meteorological datasets have a 1° horizontal resolution, divided into 23 vertical pressure levels. The maximum modeling height was set at 10,000 m above ground level. Trajectories were calculated backwards in 30 min intervals from the sampling location (49.99°N, 8.23°E). To assess a species-specific trajectory length, we followed Landolt-Börnstein (1988), which estimates the residence time of a particle based on its aerodynamic diameter. The aerodynamic diameter of a particle is defined as the diameter of a sphere with a density of 1 g cm^−3^ and the same aerodynamic properties as the particle and is primarily dependent on the particle shape and density. For reasons of simplicity, we used the lower bound of actual diameter, as both species have more or less spherical shape with width to length ratios of over 0.9 and furthermore the cell density of pollen is known to be variable, dependent on pollen age and humidity levels (http://www.pollenwarndienst.at/DE/de/allergie-infos/fuer-aerobiologen/pollenatlas.html). For ragweed, Harrington and Metger (1963) report densities for fresh pollen that ranged from 1.28 to 1.05 g cm^−3^ at 100 and less than 52% relative humidity, respectively, and a density of 0.84 g cm^−3^ for dried pollen at 52% relative humidity. This resulted in back-trajectory run times of 27 h for mugwort (20 µm) and 32 h for ragweed (18 µm) during the flowering period. Outside the flowering season, the run times were arbitrarily set to 72 h to account for potential long-range transport from southern Europe and the transport of damaged or aged pollen of smaller aerodynamic diameters.

## Results and discussion

### Seasonal variation and comparison of the pollination periods

**Fig. 1:**
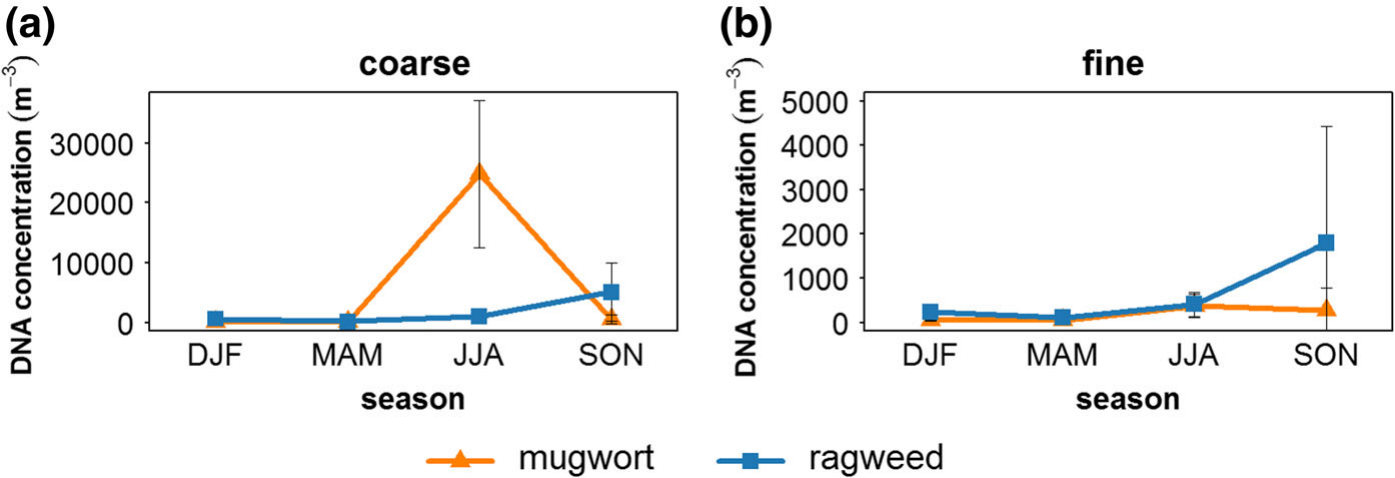
Sum of the number of DNA copies per cubic meter of air for each meteorological season averaged over the 5 years of sampling (2006–2010): Results are shown for ragweed and mugwort within the **a** coarse particle samples and the **b** fine particulate matter samples. DJF (winter: December, January, February); MAM (spring: March, April, May); JJA (summer: June, July, August); SON (fall: September, October, November). The *error bars* correspond to the standard deviation between the years

**Fig. 2:**
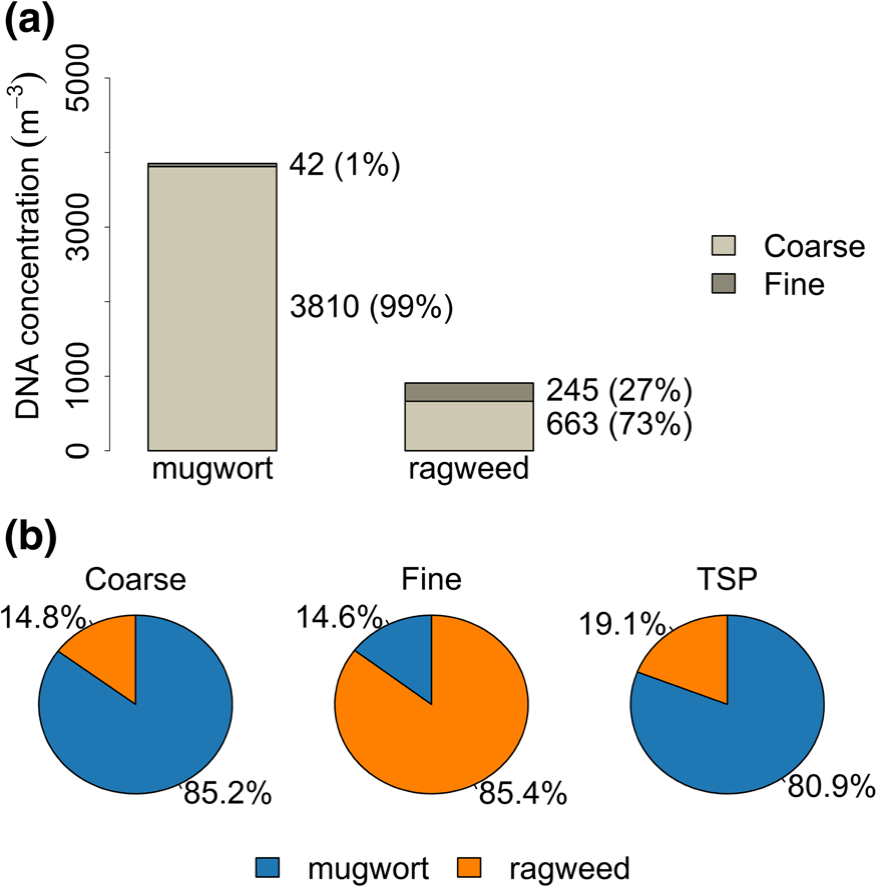
Comparison of the ragweed and mugwort DNA concentrations within their characteristic pollination periods (ragweed: mid-August to end of September, mugwort: mid-July to end of August): **a** The number of DNA copies per cubic meter of air in the coarse and fine fraction averaged over all analyzed air samples (2006–2010) that lay within the characteristic pollination periods, **b** The relative proportions of the ragweed and mugwort copies per cubic meter of air shown for the coarse and fine fractions as well as TSP (coarse + fine) calculated using the values displayed in Fig. 2a

During the 5-year sampling period from 2006 to 2010 in Mainz, mugwort as well as ragweed DNA was successfully quantified from air filters on which a mixture of urban and rural continental boundary layer air masses was collected. In total, 89 filter pairs, each consisting of a coarse and a fine particulate matter filter, were analyzed for mugwort and 67 filter pairs for ragweed. Details are shown in Table 1 listing the number of analyzed filters for each year next to the number of filters with quantifiable DNA concentrations. In Fig. 1, we show the seasonal variations in atmospheric concentrations in the coarse (1a) and fine (1b) size fractions separately, whereas in Fig. 2, we compare plant-specific pollination periods and show both results for absolute concentrations (2a) and a comparison of the relative proportions (2b). It is important to remember, when viewing Fig. 2, that the pollination periods of both plants differ, so we are comparing time periods that only overlap by half a month.

As intact mugwort pollen is spherical and 20–26 µm in diameter while ragweed pollen has a size of 18–22 µm (Table ESM1), their resulting aerodynamic diameters will lie well above the 3-µm sampler size separation between the coarse and fine fractions. Therefore, DNA from intact pollen grains will only be found in the coarse particle fraction along with plant fragments with aerodynamic diameters above the cutoff. Unsurprisingly, our data indicate that the process of pollen release is the largest contributor to the atmospheric DNA content. As can be seen in Fig. 1a, both mugwort and ragweed show their by far highest coarse fraction concentrations within their characteristic pollination periods. Mugwort is seen to peak in summer, while ragweed peaks in fall (pollination periods: mugwort: mid-July to August, ragweed: mid-August to September, see Figure ESM1 for more detail). Nonetheless, it is also possible that coarse fraction plant tissue is also released along with pollen, meaning that the DNA concentrations need not exclusively stem from pollen.

As presented in Fig. 2a, b (TSP) the mugwort concentrations within its pollination period are in total roughly four to five times as high as those for ragweed. Furthermore, when comparing the years individually the mugwort concentration in 2010 even reached the 20-fold concentration of ragweed (Figure ESM2). These observations are consistent with mugwort being far more abundant in Germany. For both plants, the proportion of DNA detected in the coarse fraction was much higher than in the fine fraction, varying between 99% for mugwort and 73% for ragweed for the coarse particles (Fig. 2a) and this again is consistent with pollen grains falling in the coarse size fraction.

However, as listed in Table 1, 92% of the coarse particle filters contained mugwort DNA, while 100% contained ragweed DNA. As the primers were equally sensitive and amplified still from 10 copies DNA, as tested in dilution series, a technical reason for this finding is less likely. For further details also see Table ESM3, where the exact copy numbers for all analyzed filter samples are provided. These findings seem to contradict the fact that mugwort is far more abundant in Germany than ragweed and thus could be expected to be found more frequently. However, for mugwort, 32 filter pairs, i.e., only 35% of the total analyzed filter pairs, lay within the specific mugwort pollination period and for ragweed 35 filter pairs and therefore over 50% lay within its characteristic pollination time. Within the mugwort-specific pollination period, from mid-July to August, 100% of the coarse filters contained quantifiable DNA. Nonetheless, quite surprisingly DNA from the far less abundant ragweed was found on all analyzed samples, regardless of size fraction or time of year, albeit at sometimes very low concentrations. This may be explained by long-range transport from southern Europe which is discussed in detail below.

In Fig. 1b the seasonal fine fraction concentrations are provided. When compared to the coarse fraction concentrations, seen in 1a, the seasonal progression of the inhalable fine fraction ragweed mirrors the coarse fraction concentrations at lower concentrations. The fine fraction mugwort concentration does show a minor peak in summer, coinciding with the mugwort pollination period; however, it is far less pronounced in comparison with ragweed.

Particles, collected in the fine particulate matter fraction, will consist of fragmented plant and pollen material. For ragweed, a frequent production of sub-pollen particles (SPPs) with a size range 0.5–4.5 µm has been observed (Bacsi et al. 2006). This SPP production is likely based on fragmentation processes, as it is known that ragweed pollen can burst under high humidity, as well as during heavy rainfall and thunderstorms, and release SPPs in great numbers (Huffman et al. 2013; Pummer et al. 2013; Steiner et al. 2015). The pollen nucleus DNA contained in some SPPs, due to their small aerodynamic diameters, is likely to be sampled predominantly in the fine particle fraction. In hydration tests with ragweed pollen, at least 35% burst within 90 min (Bacsi et al. 2006). For mugwort to our knowledge, no similar experiments have been conducted. It should also be noted that DNA from fragmented tissue material may be degraded by exposure to atmospheric photooxidants and thus might even be underestimated (Després et al. 2007).

While only 46% of the fine filter samples had quantifiable mugwort DNA, all analyzed fine filters contained ragweed DNA (Table 1). Even within its pollination period, mugwort was only found on 53% of the fine filters despite being far more abundant in the local surrounding than ragweed, which was detected in all fine particle filters. Furthermore, during the pollination period 27% of the total ragweed DNA concentration was found in the inhalable fine particulate fraction (Fig. 2a). Comparing the two species, the proportions of the highly allergenic ragweed to the native mugwort are reversed in respect to the coarse fraction with ragweed constituting 85.4% in the fine fraction (Fig. 2b). These results, therefore, firstly imply that mugwort pollen grains are far more stable than ragweed pollen grains. Secondly and more importantly, along with the continual presence of ragweed in both size fractions these observations may be important factors explaining the high allergic potential of ragweed and the high sensitization rates observed among populations exposed to ragweed, as SPPs can reach and accumulate in the alveoli of the lungs and therefore might be promoting and enhancing allergic reactions (Mücke and Lemmen 2008).

### Annual variation and the influence of meteorology

As illustrated by the large error bars seen in Fig. 1a, large variation in measured DNA concentrations was observed between the analyzed years. When comparing the DNA concentration patterns between the individual years in more detail (Figure ESM2), it becomes evident that the highest detected mugwort TSP DNA concentration for single filter samples during its pollination period varies between ~4000 copies per m^3^ in 2006 and the fivefold amount of ~19,000 copies per m^3^ in 2009. For ragweed, there is a similar picture. Between the different years, the TSP DNA concentration maxima during the pollination period ranged between ~6500 copies per m^3^ (2008) and ~500 copies per m^3^ (2010). This supports the findings of Straka (1975) that yearly pollen concentrations can vary enormously in strength. The local pollen amount is on the one hand influenced by the number, health, and size of the producing plants and on the other hand by the general growing conditions, i.e., climate and habitat characteristics (Fumanal et al. 2007). Although the main pollination season was generally more pronounced in the coarse particle fraction for both weeds, in 2008 and 2009 the fine particle filter samples display high DNA concentrations for ragweed within the pollination time, resulting in several high values from the middle of September until the middle of October 2008 with a maximum of ~2700 copies per m^3^.

Due to the continuous spread of ragweed in Europe and especially within Germany, one could expect an increase in ragweed presence (Alberternst et al. 2006; Dahl et al. 1999; Jäger 2000; Mandrioli et al. 1998; Prank et al. 2013). However, within the Mainz air filter samples, this spread is not reflected by an increase in the DNA concentration over the 5-year measurement period. With the increasing public awareness of the spread of ragweed, a greater amount of ragweed plants was detected over the last decade. In Germany, it is not compulsory to remove ragweed plants. Therefore, combative measures are locally confined and sporadic. Nonetheless, these measures might also add to the variability and counteract a potential increase in ragweed DNA amount in the observation period from 2006 to 2010. As another explanation for the annual DNA variation observed for both weeds, one could discuss possible competitive behavior between the species, influences by meteorological conditions or long-distance transport playing a substantial role.

**Fig. 3:**
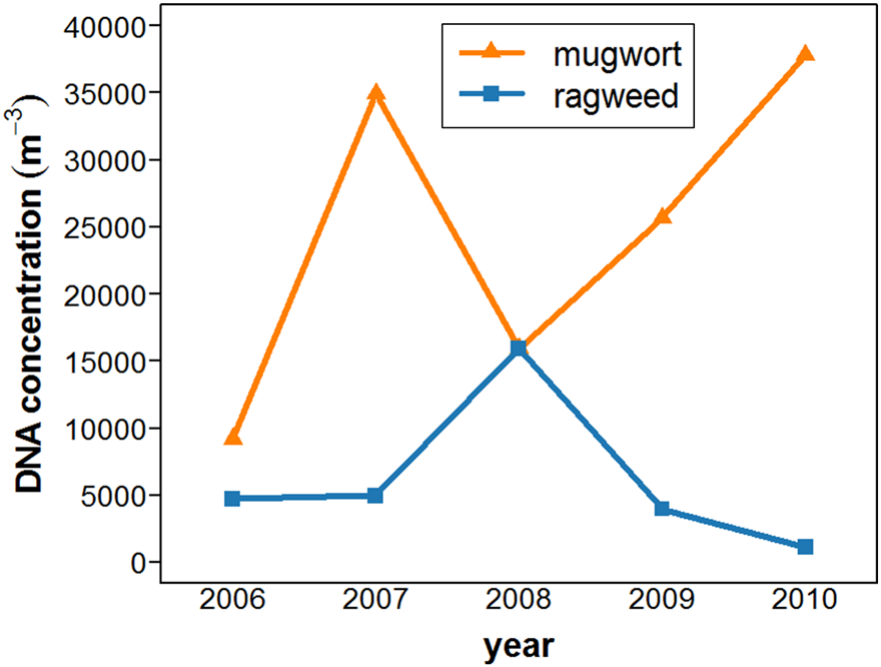
Comparison of the mugwort and ragweed sums of TSP DNA copies per cubic meter of air for all years of sampling

Figure 3 shows an almost inverse behavior of mugwort and ragweed throughout the 5 years. This could point to a possible competitive behavior between both plants for certain habitats as both weeds prefer similar growing conditions (Barney and Di Tommaso 2003; Bassett and Crompton 1975; see Table ESM1). However, the direct observation of the plant behavior makes competition effects less likely. Ragweed prefers heavily disturbed areas like agricultural fields, while mugwort avoids these disturbed areas. And even if competition takes place for a short period, it may have no effect on the long-term occupation of that habitat.

The onset and duration of characteristic pollination seasons also vary depending on different physical influences like seasonal climate, and meteorological factors (Dahl et al. 2013). Especially meteorological factors like precipitation, relative humidity, temperature, and wind direction as well as speed are known to have a strong influence on the life cycles of mugwort and ragweed, their successful pollen grain production, release and distribution (Kaminski et al. 2010; Kasprzyk 2006; Peternel et al. 2008; Puc 2006). As shown in Figure ESM3, the linear regression analysis of the meteorological data with mugwort or ragweed DNA concentration in the coarse fraction produced no significant results true for all years. This is likely due to a too simplistic approach in the linear regression analysis. Firstly, it would require an immediate effect of a meteorological factor on the atmospheric concentrations. A temporal lag in the influence is probable. Nonetheless, the DNA concentrations of individual years do show high absolute Pearson values to differing meteorological factors. Therefore, we have presented the results, but make no attempt at in-depth interpretation. Preliminary results of a timeline analysis of the meteorological factors in the time period leading up to the pollination peak suggest that the temperature profile plays a key role in the onset of pollination of both species. Furthermore, there is evidence that implies that precipitation may play a role in the DNA concentration within the fine fraction. These observations are, however, preliminary and will be subject of future analysis.

### Wind back trajectories and long-range transport

For ragweed the concentrations in all samples taken in November and December reached approximately 5–42%, (27–1085 copies per m^3^) of maximum air mass sample concentrations of the same years. In each year, the analyzed November and December samples lay well after the first temperature drop below 0 °C at the sampling location (earliest freezing temperatures on 15th of October in 2009 and latest on November 23, 2008); this should rule out the pollen release in the sampling region as a source of the DNA concentrations (Buttenschøn et al. 2009). The presence may also be due to emission related to the degradation process; however, in the same samples mugwort only reaches 0–1.8% (0–309 copies per m^3^) of its annual maximum concentration. Again, the far higher local mugwort abundancy would lead to higher concentrations if degradation was the main contributor.

As locally—emitted pollen and degrading plant matter seem unlikely as a predominant source for the high wintertime concentrations of ragweed, long-range transport from southern Europe may be a viable explanation. Pollen grains in the size range around 20 µm can be transported up to 1000 km into areas where they are not native, and for ragweed long-distance transport events have been reported in the past (Buttenschøn et al. 2009; Kaminski et al. 2010; Mandrioli et al. 1998; Piotrowska and Weryszko-Chmielewska 2006; Rousseau 2003; Smith et al. 2008; Stach et al. 2007; Zink et al. 2012). In Germany the pollen has been shown to originate from distant areas such as Slovakia, Hungary, Northern Italy, or Southern France (Smith et al. 2008; Zink et al. 2012). Other studies even report pollen reaching the UK and Denmark from southern France and Hungary (de Weger et al. 2016; Sommer et al. 2015). Furthermore, Grewling et al. (2016) were also able to show that the main allergen Amb a 1 maintained its immunoreactivity after a hypothesized long-range transport in both a size range above and below 10 µm aerodynamic diameter.

**Fig. 4:**
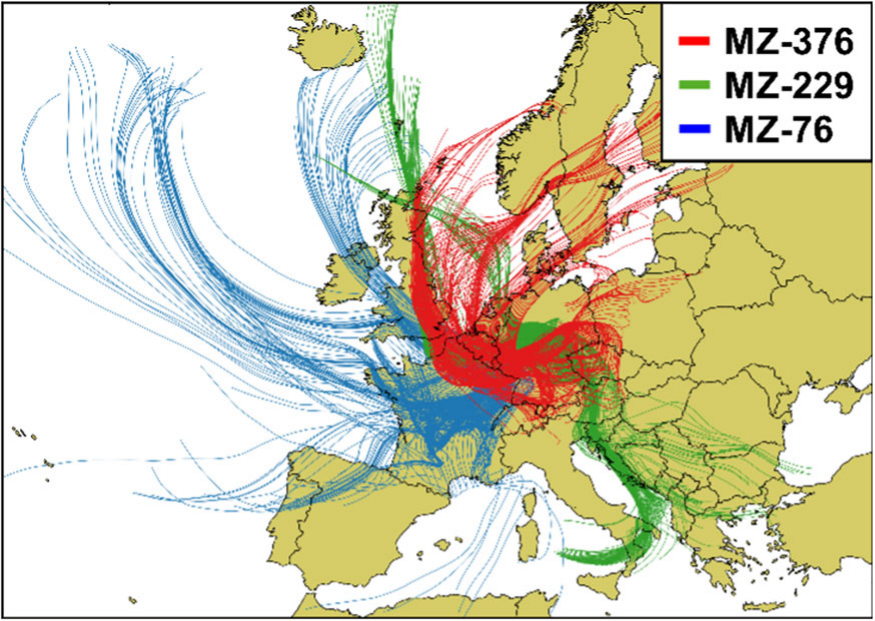
Back trajectories calculated for fall–winter periods for mugwort (**a**) and ragweed (**b**): **a** 2009 of filter sample MZ 297 (2009-11-03 to 2009-11-10; *red colored*); 2010 of filter sample MZ 376 (2010-11-23 to 2010-11-30; *green colored*); **b** 2006 of filter sample MZ 76 (2006-12-07 to 2006-12-14; *blue colored*); 2008 of filter sample MZ 229 (2008-12-11 to 2008-12-18; *green colored*); 2010 of filter sample MZ 376 (2010-11-23 to 2010-11-30; *red colored*). Trajectories were calculated using the program Hysplit

In Fig. 4, we show the calculated back trajectories for two samples with the highest wintertime ragweed concentrations (MZ-76 from 2006 (blue) and MZ-229 from 2008 (green) with TSP concentrations 1085 and 896 copies per m^3^, respectively) along with the sample with the lowest measured wintertime concentration (MZ-376 from 2010 (red) with a TSP concentration of 27 copies per m^3^). As can be seen, the back trajectories for MZ-76 seen in blue predominately stem from a southwesterly region in respect to our sampling location, from mid and southern France including the Rhône valley known for its extensive ragweed population (Thibaudon et al. 2014). Furthermore, data obtained from the RNSA (Réseau National de Surveillance Aérobiologique) for Avignon, a potential emission source, show concentrations of 0.53–1.06 grains m^−3^ 1 week prior to our sampling, which would account for the duration of the transport process from southern France. The back trajectories calculated for MZ-229, seen in green, mainly stem from a southeasterly direction, passing over the eastern Adriatic coast and to some extent the Pannonian Plain both known for their very extensive ragweed stock (Buttenschøn et al. 2009; Skjøth et al. 2010). In contrast, the back trajectories calculated for MZ-376 seen in red, with a low concentration of ragweed DNA, mainly stem from a north to northwesterly direction, with the most southern trajectories stemming from the Alps. These results strengthen the possibility of long-range transport from southern Europe as an explanation for the high wintertime concentrations.

If long-range transport of ragweed does play a significant role for the wintertime concentrations, reaching up to 42% of the annual maximum, it should also have a significant influence during the pollination season. Makra et al. (2006) report that ragweed alone makes up 47.3% of the annually measured pollen in Szeged in Hungary. Their measurements encompassed 24 taxa, including mugwort, which was shown to have an annual pollen concentration peak roughly of an order of magnitude smaller than that of ragweed. Next to possible differences in pollen stability leading to SPP production, high emission rates from southern Europe may also be a feasible alternative explanation for the high fine fraction concentrations, which could be caused by rupturing of the pollen during transport at high altitude. Large differences in emission rates between mugwort and ragweed would furthermore explain why mugwort is not as prominent in the fine fraction, as far less pollen is subjected to the environmental stresses of long-range transport.

## Conclusions

Within this study, we successfully quantified DNA of the allergenic weeds mugwort and ragweed over a 5-year period in Mainz, Germany. For both weeds, we could demonstrate that their DNA concentrations are higher within the coarse particle fraction, containing particles with aerodynamic diameters larger than ~3 µm. This is most probably due to the plants’ pollen falling in this fraction, with actual diameters of ~20 µm. Furthermore, there was a large variation between the DNA abundancy observed each year, which may be primarily explained by differing meteorological conditions. We could also demonstrate that, within the coarse fraction, more mugwort was present than ragweed, which coincides with mugwort being far more abundant within the local flora than ragweed.

Interestingly, the situation was reversed in the inhalable fine particle fraction (<~3 µm), with more ragweed DNA present than mugwort DNA. An explanation for this finding might be that ragweed pollen is less stable and has a higher tendency to burst under humid conditions, thereby producing SPPs, which are smaller in size and thus accumulate on the fine particle filter. We also observe high ragweed DNA concentrations outside the pollination season. Back-trajectory analysis pointed to long-distance transport from southern Europe, where the pollen emissions of ragweed are reported to far outweigh those of mugwort. This may be an alternative explanation of the high fine fraction concentrations, in general, as the pollen may be rupturing during long-range transport.

The high fine fraction concentrations may also be a factor contributing to the allergenic potential of ragweed and high sensitization rates seen among the population as small particles will spread further, heightening exposure, and furthermore penetrate deeper in the respiratory system. The influence of long-distance transport implies that not only the local vegetation must be taken into account when discussing possible health risks but also the pollen and pollen fragments reaching Germany from surrounding countries.

## Electronic supplementary material

Below is the link to the electronic supplementary material.

Supplementary material 1 (DOCX 78 kb)

Supplementary material 2 (PNG 34 kb)

Supplementary material 3 (PNG 566 kb)

Supplementary material 4 (PNG 1196 kb)

Supplementary material 5 (PNG 985 kb)
