# Supplementary material for: Allergenic Asteraceae in air particulate matter: quantitative DNA analysis of mugwort and ragweed
Source: Aerobiologia (Bologna). 2017 Jun 6;33(4):493–506. doi: 10.1007/s10453-017-9485-3 (PMC5674138; doi:10.1007/s10453-017-9485-3)
Supplement: Supplementary file 1 — Supplementary material 1 (DOCX 78 kb) [file 10453_2017_9485_MOESM1_ESM.docx]

**Allergenic Asteraceae in air particulate matter: DNA-based analysis of mugwort and ragweed**

**Aerobiologia**

I. Müller-Germann^1,2^, D.A. Pickersgill^1,3^, H. Paulsen^3^, B. Alberternst^4^, U. Pöschl^1^, J. Fröhlich-Nowoisky ^1^ and V. R. Després^1,3^

^1^Max Planck Institute for Chemistry, Biogeochemistry and Multiphase Chemistry Departments, Hahn-Meitner-Weg 1, D-55128 Mainz, Germany

^2^Geosciences, Johannes Gutenberg University, Joh.-Joachim-Becher-Weg 21, D-55128 Mainz, Germany

^3^Molecular Physiology, Johannes Gutenberg University, Joh.-von-Müller-Weg 6, D-55099 Mainz, Germany

^4^Working Group Biodiversity and Landscape Ecology, Hinter’m alten Ort 9, D-61169 Friedberg, Germany

**Corresponding author**

| Viviane R. Després  Phone/Fax: 0049-6131-392-4203/3787  Email: [despres@uni-mainz.de](mailto:despres@uni-mainz.de) |
| --- |

**Supplementary Online Material**

**Meteorological influences**

Interestingly, some years seem to show in total more significant correlations than others, e.g., for fine particles in ragweed in the year 2006 the temperature seems to correlate negatively with the DNA concentration, while wind speed criteria correlate positively. In the coarse particle fraction these correlations are not supported. For mugwort, however, the year 2008 sticks out. Here in the coarse particle fraction criteria describing the influence of relative humidity and precipitation, correlate either positively or negatively. Observations by other authors, who found e.g. influence by wind speed on the pollen dispersal, cannot be supported strongly with the presented data (Dahl et al. 2013). On the other hand, the findings do support the results of studies dealing with the comparison of meteorological data and mugwort and ragweed pollen concentration, which also found no continuous trends for the correlation (Kaminski et al. 2010; Puc 2006) . However, meteorological parameters should still influence mugwort and ragweed life cycles. It might be that these correlations can only be discovered using a smaller time scale. Furthermore, it is also possible that there is a temporal lag in the influence of meteorological parameters, which is not taken into account in our analysis.

**Supplementary Figure Titles**

**Fig. ESM1** Primer binding sites for mugwort and ragweed in the ITS region

**Fig. ESM2** Mugwort (a) and ragweed (b) DNA concentration in air from 2006 to 2010. DNA amount is given in copies per cubic meter of air: m^-3^; m^-3^x1000 for coarse and fine particle filters for each analyzed filter sample. The sum for the DNA concentration on coarse and fine particle filters gives the DNA concentration of the total air mass. Gaps in the x-axis represent non-analyzed time periods in late spring/early summer and late fall

**Fig. ESM3** Pearson heat map depicting the Pearson values (green +1 to red -1) between copy numbers of *Ambrosia* and *Artemisia* in the TSP, coarse and fine fractions and meteorological factors. For each year only the samples were factored into the analysis that lay before the peak coarse fraction copy numbers, which should correspond to the maximum pollination time point. Meteorological factors analysed were temperature (T in °C), relative humidity (RH in %), wind speed (WS), precipitation (Precip), the sum of precipitation duration as well as average precipitation strength

**Supplementary Tables**

**Table ESM1** Comparison of mugwort and ragweed plant characteristics (n.k. = not known)

|  |  | **Ragweed (*Ambrosia artemisiifolia*)** | |  | **Mugwort (*Artemisia vulgaris*)** | |
| --- | --- | --- | --- | --- | --- | --- |
| **Origin** |  | North America and Canada | e.g., Gadermaier et al. (2004) |  | Europe | Barney and Di Tommaso (2003) and references therein |
| **Habitat** |  | Human disturbed open habitats, roadsides | Bassett and Crompton (1975) |  | Wastelands, roadsides | Holm et al. (1997) |
| **Climate/soil conditions** |  | Warm climate, grows in a wide range of soil types, prefers slightly acidic, sandy and muddy loam soils | Bassett and Crompton (1975), own observations |  | Wide temperature range, moist soils | Barney and Di Tommaso (2003) |
| **European distribution pattern** |  | Centre: Northern Italy, southern France, Carpathian Basin  Spreading northwards | Dahl et al. (1999); Makra et al. (2004)  Dahl et al. (1999) |  | Northern hemisphere and Mediterranean basin | Gadermaier et al. (2004) |
| **Generation cycle** |  | Annual | Peternel et al. (2005) |  | Rhizomatous perennial | Barney and Di Tommaso (2003) |
| **Seed production** |  | 3000-62000 seeds depending on plant size | Dahl et al. (1999) |  | 200 000 seeds per plant | Pawlowski et al. (1968) |
| **Main pollination season** |  | August-onset of frost depending on local climate  some plants start to flower at the end of June | Buttenschøn et al. (2009); Dahl et al. (1999), own observations |  | End of July-end of August, can last longer in warmer regions | Gadermaier et al. (2004); Wopfner et al. (2005) |
| **Pollen grain size** |  | 18- 22 µm | Gadermaier et al. (2004) |  | 20 – 26 µm | Hayat et al. (2009) |
| **Pollen production** |  | 10^9^-2,5x10^9^ | Fumanal et al. (2007); Laaidi et al. (2003b) |  | 3,19 x 10^9^ | Grewling et al. (2012); Piotrowska (2008) |
| **Prevalence in atopic individuals** |  | Europe: 35-80 %  North America: 45 % | Bundesamt für Gesundheit (2005); Wopfner et al. (2005) |  | Europe: 14 %  North America: n.k. | Spieksma et al. (1980) |

**Table ESM2** Start-up and mounting blank measurements (+ DNA detected on blank sample; - no DNA detected; n.a. not analyzed)

| **Description** | **Sampling Date** | **Sampling period (in days)** | ***Artemisia vulgaris*** | ***Ambrosia artemisiifolia*** |
| --- | --- | --- | --- | --- |
| MZ 48a | 2006-08-02 | Start-up blank | - | - |
| MZ 48b | 2006-08-02 | Start-up blank | - | - |
| MZ 49a | 2006-08-02 | Mounting blank | + | - |
| MZ 49b | 2006-08-02 | Mounting blank | - | - |
| MZ 56a | 2006-09-11 | Start-up blank | - | - |
| MZ 56b | 2006-09-11 | Start-up blank | - | - |
| MZ 58a | 2006-09-11 | Mounting blank | - | - |
| MZ 58b | 2006-09-11 | Mounting blank | - | - |
| MZ 132a | 2007-09-06 | Start-up blank | - | - |
| MZ 132b | 2007-09-07 | Start-up blank | - | - |
| MZ 133a | 2007-09-07 | Mounting blank | - | - |
| MZ 133b | 2007-09-07 | Mounting blank | - | - |
| MZ 140a | 2007-10-11 | Start-up blank | - | - |
| MZ 140b | 2007-10-11 | Start-up blank | - | - |
| MZ 141a | 2007-10-11 | Mounting blank | - | - |
| MZ 141b | 2007-10-11 | Mounting blank | - | - |
| MZ 201a | 2008-08-07 | Start-up blank | + | - |
| MZ 201b | 2008-08-07 | Start-up blank | - | - |
| MZ 202a | 2008-08-07 | Mounting blank | - | - |
| MZ 202b | 2008-08-07 | Mounting blank | - | - |
| MZ 207a | 2008-09-04 | Start-up blank | - | - |
| MZ 207b | 2008-09-04 | Start-up blank | - | - |
| MZ 208a | 2008-09-04 | Mounting blank | - | - |
| MZ 208b | 2008-09-04 | Mounting blank | - | - |
| MZ 213a | 2008-10-02 | Start-up blank | - | - |
| MZ 213b | 2008-10-02 | Start-up blank | - | - |
| MZ 214a | 2008-10-02 | Mounting blank | - | - |
| MZ 214b | 2008-10-02 | Mounting blank | - | - |
| MZ 271a | 2009-07-14 | Start-up blank | - | - |
| MZ 271b | 2009-07-14 | Start-up blank | - | - |
| MZ 272a | 2009-07-14 | Mounting blank | - | - |
| MZ 272b | 2009-07-14 | Mounting blank | - | - |
| MZ 277a | 2009-08-11 | Start-up blank | - | - |
| MZ 277b | 2009-08-11 | Start-up blank | - | - |
| MZ 278a | 2009-08-11 | Mounting blank | - | - |
| MZ 278b | 2009-08-11 | Mounting blank | - | - |
| MZ 281a | 2009-09-01 | Start-up blank | - | - |
| MZ 281b | 2009-09-01 | Start-up blank | - | - |
| MZ 282a | 2009-09-01 | Mounting blank | - | - |
| MZ 282b | 2009-09-01 | Mounting blank | - | - |
| MZ 290a | 2009-10-13 | Start-up blank | - | - |
| MZ 290b | 2009-10-13 | Start-up blank | - | - |
| MZ 291a | 2009-10-13 | Mounting blank | - | - |
| MZ 291b | 2009-10-13 | Mounting blank | - | - |
| MZ 346a | 2010-07-06 | Start-up blank | - | n.a. |
| MZ 346b | 2010-07-06 | Start-up blank | - | n.a. |
| MZ 347a | 2010-07-06 | Mounting blank | - | n.a. |
| MZ 347a | 2010-07-06 | Mounting blank | - | n.a. |
| MZ 353a | 2010-08-10 | Start-up blank | - | - |
| MZ 353b | 2010-08-10 | Start-up blank | - | - |
| MZ 354a | 2010-08-10 | Mounting blank | - | - |
| MZ 354b | 2010-08-10 | Mounting blank | - | - |
| MZ 358a | 2010-08-31 | Start-up blank | - | - |
| MZ 358b | 2010-08-31 | Start-up blank | - | - |
| MZ 359a | 2010-08-31 | Mounting blank | - | - |
| MZ 359b | 2010-08-31 | Mounting blank | - | - |
| MZ 365a | 2010-10-05 | Start-up blank | - | - |
| MZ 365b | 2010-10-05 | Start-up blank | - | - |
| MZ 366a | 2010-10-05 | Mounting blank | - | - |
| MZ 366b | 2010-10-05 | Mounting blank | - | - |
| Ex Blk | 2008-08-06 | Extraction blank | - | - |
| Ex Blk | 2008-08-07 | Extraction blank | - | - |
| Ex Blk | 2008-08-19 | Extraction blank | - | - |
| Ex Blk | 2008-09-17 | Extraction blank | - | - |
| Ex Blk | 2008-11-12 | Extraction blank | - | - |
| Ex Blk | 2008-12-29 | Extraction blank | - | - |
| Ex Blk | 2009-11-03 | Extraction blank | - | - |
| Ex Blk | 2009-11-04 | Extraction blank | - | - |
| Ex Blk | 2009-11-12 | Extraction blank | - | - |
| Ex Blk | 2010-09-23 | Extraction blank | - | - |
| Ex Blk | 2010-09-23 | Extraction blank | - | - |
| Ex Blk | 2010-11-17 | Extraction blank | - | - |

**Table ESM3** DNA copies per cubic meter of air; m^3^ are given for mugwort (a) and for ragweed (b) in the sampling period from 2006-2010

1. **Mugwort**

| **Filter ID** | **Sampling period (2006)** | **Sampled air volume [m³]** | **Coarse filters [m^­­-^³]** | **Fine filters [m­­^­-^³]** | **Air masses [m^­­-^³]** |
| --- | --- | --- | --- | --- | --- |
| MZ 24 | 05-18 / 05-22 | 1703 | 95 | 99 | 194 |
| MZ 41 | 07-04 / 07-11 | 3019 | 17 | 298 | 315 |
| MZ 42 | 07-11 / 07-14 | 1315 | 34 | 86 | 120 |
| MZ 43 | 07-14 / 07-17 | 1288 | 63 | 89 | 152 |
| MZ 47 | 07-26 / 08-02 | 3032 | 8 | 39 | 47 |
| MZ 50 | 08-02 / 08-09 | 2931 | 2247 | 0 | 2247 |
| MZ 51 | 08-09 / 08-16 | 3015 | 3849 | 193 | 4042 |
| MZ 52 | 08-16 / 08-23 | 3056 | 2197 | 0 | 2197 |
| MZ 53 | 08-23 / 08-30 | 3054 | 306 | 21 | 327 |
| MZ 54 | 08-30 / 09-06 | 3087 | 179 | 0 | 179 |
| MZ 55 | 09-06 / 09-11 | 2139 | 121 | 0 | 121 |
| MZ 59 | 09-11 / 09-18 | 2916 | 26 | 0 | 26 |
| MZ 60 | 09-18 / 09-25 | 3073 | 32 | 0 | 32 |
| MZ 61 | 09-25 / 10-02 | 3063 | 24 | 2 | 26 |
| MZ 62 | 10-02 / 10-09 | 3033 | 12 | 1 | 13 |
| MZ 76 | 12-07 / 12-14 | 3058 | 5 | 36 | 41 |
| **Filter ID** | **Sampling period (2007)** | **Sampled air volume [m³]** | **Coarse filters [m^­­-^³]** | **Fine filters [m­­^­-^³]** | **Air masses [m^­­-^³]** |
| MZ 108 | 05-10 / 05-17 | 3025 | 2 | 131 | 133 |
| MZ 121 | 07-05 / 07-12 | 3034 | 58 | 9 | 67 |
| MZ 122 | 07-12 / 07-19 | 3076 | 301 | 0 | 301 |
| MZ 123 | 07-19 / 07-26 | 3001 | 2114 | 103 | 2217 |
| MZ 126 | 07-26 / 08-02 | 3068 | 5404 | 38 | 5442 |
| MZ 127 | 08-02 / 08-09 | 3064 | 7646 | 16 | 7662 |
| MZ 128 | 08-09 / 08-16 | 3065 | 17056 | 22 | 17078 |
| MZ 129 | 08-16 / 08-23 | 3038 | 1701 | 9 | 1710 |
| MZ 130 | 08-23 / 08-30 | 3033 | 776 | 0 | 776 |
| MZ 131 | 08-30 / 09-06 | 3025 | 25 | 0 | 25 |
| MZ 134 | 09-06 / 09-13 | 3042 | 6 | 0 | 6 |
| MZ 135 | 09-13 / 09-20 | 3043 | 364 | 0 | 364 |
| MZ 136 | 09-20 / 09-27 | 3072 | 27 | 0 | 27 |
| MZ 137 | 09-27 / 10-04 | 3046 | 15 | 0 | 15 |
| MZ 138 | 10-04 / 10-11 | 3056 | 11 | 0 | 11 |
| MZ 153 | 12-06 / 12-13 | 3052 | 276 | 33 | 309 |
| **Filter ID** | **Sampling period (2008)** | **Sampled air volume [m³]** | **Coarse filters [m^­­-^³]** | **Fine filters [m­­^­-^³]** | **Air masses [m^­­-^³]** |
| MZ 181 | 04-24 / 05-01 | 3046 | 4 | 23 | 27 |
| MZ 194 | 07-03 / 07-10 | 3006 | 13 | 82 | 95 |
| MZ 197 | 07-10 / 07-17 | 2995 | 406 | 3 | 409 |
| MZ 198 | 07-17 / 07-24 | 3082 | 20 | 8 | 28 |
| MZ 199 | 07-24 / 07-31 | 3039 | 1140 | 0 | 1140 |
| MZ 200 | 07-31 / 08-07 | 3078 | 12333 | 0 | 12333 |
| MZ 203 | 08-07 / 08-14 | 3010 | 2058 | 0 | 2058 |
| MZ 204 | 08-14 / 08-21 | 3147 | 260 | 0 | 260 |
| MZ 205 | 08-21 / 08-28 | 2969 | 85 | 0 | 85 |
| MZ 206 | 08-28 / 09-04 | 3106 | 1 | 0 | 1 |
| MZ 209 | 09-04 / 09-11 | 3069 | 1 | 51 | 52 |
| MZ 210 | 09-11 / 09-18 | 1687 | 6 | 0 | 6 |
| MZ 211 | 09-18 / 09-25 | 3050 | 8 | 0 | 8 |
| MZ 212 | 09-25 / 10-02 | 3080 | 6 | 0 | 6 |
| MZ 215 | 10-02 / 10-09 | 2994 | 16 | 0 | 16 |
| MZ 229 | 12-11 / 12-18 | 3125 | 50 | 60 | 110 |
| **Filter ID** | **Sampling period (2009)** | **Sampled air volume [m³]** | **Coarse filters [m^­­-^³]** | **Fine filters [m­­^­-^³]** | **Air masses [m^­­-^³]** |
| MZ 261 | 05-19 / 05-26 | 3078 | 0 | 0 | 0 |
| MZ 269 | 06-30 / 07-07 | 3171 | 418 | 48 | 466 |
| MZ 270 | 07-07 / 07-14 | 3021 | 497 | 0 | 497 |
| MZ 273 | 07-14 / 07-21 | 3065 | 1531 | 0 | 1531 |
| MZ 274 | 07-21 / 07-28 | 2942 | 3339 | 18 | 3357 |
| MZ 275 | 07-28 / 08-04 | 3119 | 19097 | 199 | 19296 |
| MZ 276 | 08-04 / 08-11 | 3057 | 1055 | 0 | 1055 |
| MZ 279 | 08-11 / 08-18 | 3016 | 406 | 0 | 406 |
| MZ 280 | 08-18 / 08-25 | 3043 | 20 | 7 | 27 |
| MZ 281 | 08-25 / 09-01 | 3056 | 4 | 0 | 4 |
| MZ 284 | 09-01 / 09-08 | 3043 | 2 | 4 | 6 |
| MZ 285 | 09-08 / 09-15 | 2986 | 0 | 0 | 0 |
| MZ 286 | 09-15 / 09-22 | 3037 | 0 | 0 | 0 |
| MZ 287 | 09-22 / 09-29 | 3030 | 44 | 84 | 128 |
| MZ 288 | 09-29 / 10-06 | 3057 | 15 | 29 | 44 |
| MZ 289 | 10-06 / 10-13 | 3015 | 97 | 0 | 97 |
| MZ 292 | 10-13 / 10-20 | 2982 | 0 | 0 | 0 |
| MZ 293 | 10-20 / 10-27 | 3045 | 0 | 0 | 0 |
| MZ 297 | 11-03 / 11-10 | 3047 | 16 | 0 | 16 |
| **Filter ID** | **Sampling period (2010)** | **Sampled air volume [m³]** | **Coarse filters [m^­­-^³]** | **Fine filters [m­­^­-^³]** | **Air masses [m^­­-^³]** |
| MZ 335 | 05-04 / 05-11 | 3031 | 31 | 0 | 31 |
| MZ 345 | 06-29 / 07-06 | 3006 | 302 | 0 | 302 |
| MZ 348 | 07-06 / 07-13 | 3120 | 345 | 0 | 345 |
| MZ 349 | 07-13 / 07-20 | 3052 | 1890 | 0 | 1890 |
| MZ 350 | 07-20 / 07-27 | 3126 | 1678 | 154 | 1832 |
| MZ 351 | 07-27 / 08-03 | 3013 | 7948 | 0 | 7948 |
| MZ 352 | 08-03 / 08-10 | 3131 | 7721 | 0 | 7721 |
| MZ 355 | 08-10 / 08-17 | 3038 | 16047 | 397 | 16444 |
| MZ 356 | 08-17 / 08-24 | 3007 | 1136 | 9 | 1145 |
| MZ 357 | 08-24 / 08-31 | 3022 | 792 | 32 | 824 |
| MZ 360 | 08-31 / 09-07 | 3020 | 99 | 0 | 99 |
| MZ 361 | 09-07 / 09-14 | 3142 | 420 | 0 | 420 |
| MZ 362 | 09-14 / 09-21 | 3042 | 120 | 0 | 120 |
| MZ 363 | 09-21 / 09-28 | 2931 | 154 | 1 | 155 |
| MZ 364 | 09-28 / 10-05 | 3294 | 88 | 0 | 88 |
| MZ 367 | 10-05 / 10-12 | 3129 | 773 | 105 | 878 |
| MZ 368 | 10-12 / 10-19 | 2978 | 89 | 0 | 89 |
| MZ 369 | 10-19 / 10-26 | 3050 | 9 | 809 | 818 |
| MZ 370 | 10-26 / 11-02 | 2939 | 0 | 28 | 28 |
| MZ 373 | 11-02 / 11-09 | 3047 | 22 | 62 | 84 |
| MZ 374 | 11-09 / 11-16 | 3019 | 0 | 0 | 0 |
| MZ 376 | 11-23 / 11-30 | 3083 | 55 | 194 | 249 |

1. **Ragweed**

| **Filter ID** | **Sampling period (2006)** | **Sampled air volume [m³]** | **Coarse filters [m^­­-^³]** | **Fine filters [m­­^­-^³]** | **Air masses [m^­­-^³]** |
| --- | --- | --- | --- | --- | --- |
| MZ 24 | 05-18 / 05-22 | 1703 | 332 | 165 | 497 |
| MZ 50 | 08-02 / 08-09 | 2980 | 101 | 58 | 159 |
| MZ 51 | 08-09 / 08-16 | 2990 | 196 | 51 | 247 |
| MZ 52 | 08-16 / 08-23 | 3081 | 274 | 41 | 315 |
| MZ 53 | 08-23 / 08-30 | 3104 | 249 | 101 | 350 |
| MZ 54 | 08-30 / 09-06 | 3137 | 2529 | 12 | 2541 |
| MZ 55 | 09-06 / 09-11 | 2157 | 126 | 35 | 161 |
| MZ 59 | 09-11 / 09-18 | 2892 | 566 | 39 | 605 |
| MZ 60 | 09-18 / 09-25 | 3033 | 176 | 100 | 276 |
| MZ 61 | 09-25 / 10-02 | 3038 | 356 | 91 | 447 |
| MZ 62 | 10-02 / 10-09 | 3008 | 408 | 126 | 534 |
| MZ 76 | 12-07 / 12-14 | 3058 | 856 | 229 | 1085 |
| **Filter ID** | **Sampling period (2007)** | **Sampled air volume [m³]** | **Coarse filters [m^­­-^³]** | **Fine filters [m­­^­-^³]** | **Air masses [m^­­-^³]** |
| MZ 108 | 05-10 / 05-17 | 3075 | 268 | 163 | 431 |
| MZ 127 | 08-02 / 08-09 | 3140 | 42 | 40 | 82 |
| MZ 128 | 08-09 / 08-16 | 3115 | 195 | 128 | 323 |
| MZ 129 | 08-16 / 08-23 | 3088 | 130 | 62 | 192 |
| MZ 130 | 08-23 / 08-30 | 3033 | 537 | 56 | 593 |
| MZ 131 | 08-30 / 09-06 | 3100 | 11 | 7 | 18 |
| MZ 134 | 09-06 / 09-13 | 3092 | 42 | 15 | 57 |
| MZ 135 | 09-13 / 09-20 | 3093 | 1513 | 63 | 1576 |
| MZ 136 | 09-20 / 09-27 | 3123 | 2186 | 119 | 2305 |
| MZ 137 | 09-27 / 10-04 | 3097 | 130 | 91 | 221 |
| MZ 138 | 10-04 / 10-11 | 3056 | 391 | 211 | 602 |
| MZ 153 | 12-06 / 12-13 | 3102 | 413 | 191 | 604 |
| **Filter ID** | **Sampling period (2008)** | **Sampled air volume [m³]** | **Coarse filters [m^­­-^³]** | **Fine filters [m­­^­-^³]** | **Air masses [m^­­-^³]** |
| MZ 181 | 04-24 / 05-01 | 2921 | 99 | 84 | 183 |
| MZ 200 | 07-31 / 08-07 | 3027 | 126 | 76 | 202 |
| MZ 203 | 08-07 / 08-14 | 2960 | 204 | 350 | 554 |
| MZ 204 | 08-14 / 08-21 | 3044 | 150 | 93 | 243 |
| MZ 205 | 08-21 / 08-28 | 2920 | 112 | 191 | 303 |
| MZ 206 | 08-28 / 09-04 | 3055 | 93 | 11 | 104 |
| MZ 209 | 09-04 / 09-11 | 3018 | 572 | 46 | 618 |
| MZ 210 | 09-11 / 09-18 | 1659 | 3841 | 2678 | 6519 |
| MZ 211 | 09-18 / 09-25 | 3025 | 4372 | 1476 | 5848 |
| MZ 212 | 09-25 / 10-02 | 2980 | 952 | 1337 | 2289 |
| MZ 215 | 10-02 / 10-09 | 2994 | 3727 | 386 | 4113 |
| MZ 229 | 12-11 / 12-18 | 3074 | 582 | 314 | 896 |
| **Filter ID** | **Sampling period (2009)** | **Sampled air volume [m³]** | **Coarse filters [m^­­-^³]** | **Fine filters [m­­^­-^³]** | **Air masses [m^­­-^³]** |
| MZ 261 | 05-19 / 05-26 | 2664 | 92 | 150 | 242 |
| MZ 276 | 08-04 / 08-11 | 2708 | 461 | 221 | 682 |
| MZ 279 | 08-11 / 08-18 | 2729 | 327 | 166 | 493 |
| MZ 280 | 08-18 / 08-25 | 2634 | 1397 | 181 | 1578 |
| MZ 281 | 08-25 / 09-01 | 2712 | 363 | 211 | 574 |
| MZ 284 | 09-01 / 09-08 | 2603 | 334 | 258 | 592 |
| MZ 285 | 09-08 / 09-15 | 2583 | 54 | 220 | 274 |
| MZ 286 | 09-15 / 09-22 | 2678 | 25 | 303 | 328 |
| MZ 287 | 09-22 / 09-29 | 2721 | 61 | 63 | 124 |
| MZ 288 | 09-29 / 10-06 | 2468 | 87 | 331 | 418 |
| MZ 289 | 10-06 / 10-13 | 2630 | 668 | 278 | 946 |
| MZ 292 | 10-13 / 10-20 | 2490 | 567 | 58 | 625 |
| MZ 293 | 10-20 / 10-27 | 2560 | 401 | 383 | 784 |
| MZ 297 | 11-03 / 11-10 | 2554 | 180 | 20 | 200 |
| **Filter ID** | **Sampling period (2010)** | **Sampled air volume [m³]** | **Coarse filters [m^­­-^³]** | **Fine filters [m­­^­-^³]** | **Air masses [m^­­-^³]** |
| MZ 335 | 05-04 / 05-11 | 2981 | 16 | 1 | 17 |
| MZ 352 | 08-03 / 08-10 | 3080 | 48 | 1 | 49 |
| MZ 355 | 08-10 / 08-17 | 2988 | 15 | 8 | 23 |
| MZ 356 | 08-17 / 08-24 | 2958 | 45 | 1 | 46 |
| MZ 357 | 08-24 / 08-31 | 2923 | 6 | 18 | 24 |
| MZ 360 | 08-31 / 09-07 | 2921 | 32 | 3 | 35 |
| MZ 361 | 09-07 / 09-14 | 3039 | 92 | 10 | 102 |
| MZ 362 | 09-14 / 09-21 | 2992 | 293 | 63 | 356 |
| MZ 363 | 09-21 / 09-28 | 2883 | 71 | 3 | 74 |
| MZ 364 | 09-28 / 10-05 | 3240 | 431 | 54 | 485 |
| MZ 367 | 10-05 / 10-12 | 3078 | 90 | 68 | 158 |
| MZ 368 | 10-12 / 10-19 | 2930 | 30 | 38 | 68 |
| MZ 369 | 10-19 / 10-26 | 3000 | 23 | 19 | 42 |
| MZ 370 | 10-26 / 11-02 | 2770 | 27 | 56 | 83 |
| MZ 373 | 11-02 / 11-09 | 2947 | 21 | 27 | 48 |
| MZ 374 | 11-09 / 11-16 | 2970 | 28 | 61 | 89 |
| MZ 376 | 11-23/ 11-30 | 3083 | 5 | 22 | 27 |

**References**

Barney, J. N., & Di Tommaso, A. (2003). The biology of Canadian weeds . 118 . Artemisia vulgaris L. *Journal of Plant Science*, *83*, 205–215.

Bassett, L. J., & Crompton, C. W. (1975). The biology of Canadian weeds. 11. Ambrosia artemisiifolia L. and A. psilostachya DC. *Canadian Journal of Plant Sciences*, *55*, 463–476.

Bundesamt für Gesundheit. (2005). Bulletin 30/05: Ambrosia-eine Pflanze, die die Gesundheit Millionen kostet.

Buttenschøn, R. M., Waldispühl, S., & Bohren, C. (2009). Guidelines for management of common ragweed, Ambrosia artemisiifolia. *Forest and Landscape*, 1–47.

Dahl, A., Galán, C., Hajkova, L., Pauling, A., Sikoparija, B., Smith, M., & Vokou, D. (2013). The Onset, Course and Intensity of the Pollen Season. In M. Sofiev & K.-C. Bergmann (Eds.), *Allergenic Pollen A Review of the Production, Release, Distribution and Health Impacts* (pp. 29–70). Dordrecht, Heidelberg, New York, London: Springer.

Dahl, A., Strandhede, S., & Wihl, J.-Å. (1999). Ragweed – An allergy risk in Sweden? *Aerobiologia*, *15*, 293–297.

Fumanal, B., Chauvel, B., & Bretagnolle, F. (2007). Estimation of pollen and seed production of common ragweed in France. *Annals of Agricultural and Environmental Medicine*, *14*, 233–236.

Gadermaier, G., Dedic, A., Obermeyer, G., Frank, S., Himly, M., & Ferreira, F. (2004). Biology of weed pollen allergens. *Current Allergy and Asthma Reports*, *4*(5), 391–400.

Grewling, L., Šikoparija, B., Skjøth, C. A., Radišić, P., Apatini, D., Magyar, D., et al. (2012). Variation in Artemisia pollen seasons in Central and Eastern Europe. *Agricultural and Forest Meteorology*, *160*, 48–59.

Hayat, M. Q., Ashraf, M., Khan, M. A., Yasmin, G., Shaheen, N., & Jabeen, S. (2009). Phylogenetic analysis of Artemisia L . ( Asteraceae ) based on micromorphological traits of pollen grains. *African Journal of Biotechnology*, *8*(23), 6561–6568.

Holm, L., Doll, J., Holm, E., Pancho, J., & Herberger, J. (1997). *World Weeds: Natural Histories and Distribution*. New York: John Wiley and Sons.

Kaminski, U., Alberternst, B., Gabrio, T., Böhme, M., Nawrath, S., & Behrendt, H. (2010). Ambrosia Pollen-Konzentrationen in Baden-Württemberg. *Umweltmedizin Forschung Praxis*, *15*(1), 6–14.

Laaidi, M., Thibaudon, M., & Besancenot, J.-P. (2003). Two statistical approaches to forecasting the start and duration of the pollen season of Ambrosia in the area of Lyon (France). *International Journal of Biometeorology*, *48*(2), 65–73.

Makra, L., Juhász, M., Borsos, E., & Béczi, R. (2004). Meteorological variables connected with airborne ragweed pollen in Southern Hungary. *International Journal of Biometeorology*, *49*(1), 37–47.

Pawlowski, F., Kapeluszny, T., Kolasa, A., & Lecyk, Z. (1968). Fertility of some species of ruderal weeds. *Annales Universitatis Mariae Curie-Sklodowska. Second Edition, Agriculture*, *22*, 221–223.

Peternel, R., Srnec, L., Hrga, I., Mitic, B., Vukusic, I., & Hrga, I. (2005). Variation in ragweed (Ambrosia artemisiifolia L.) pollen concentration in central Croatia, 2002-2003. *Annals of Agricultural and Environmental Medicine*, *12*, 11–16.

Piotrowska, K. (2008). Pollen production in selected species of anemophilous plants. *Acta Agrobotanica*, *61*(1), 41–52.

Puc, M. (2006). Ragweed and mugwort pollen in Szczecin, Poland. *Aerobiologia*, *22*, 67–78.

Spieksma, F. T. M., Charpin, H., Nolard, N., & Stix, E. (1980). City spore concentrations in the European Economic Community ( EEC ) IV. Summer weed pollen (Rumex. Plantago. Chenopodiaceae. Artemisia), 1976 and 1977. *Clinical Allergy*, *10*, 319–329.

Wopfner, N., Gadermaier, G., Egger, M., Asero, R., Ebner, C., Jahn-Schmid, B., & Ferreira, F. (2005). The spectrum of allergens in ragweed and mugwort pollen. *International Archives of Allergy and Immunology*, *138*(4), 337–346.
