# Supplementary figures and images for: Allergenic Asteraceae in air particulate matter: quantitative DNA analysis of mugwort and ragweed
Source: Aerobiologia (Bologna). 2017 Jun 6;33(4):493–506. doi: 10.1007/s10453-017-9485-3 (PMC5674138; doi:10.1007/s10453-017-9485-3)

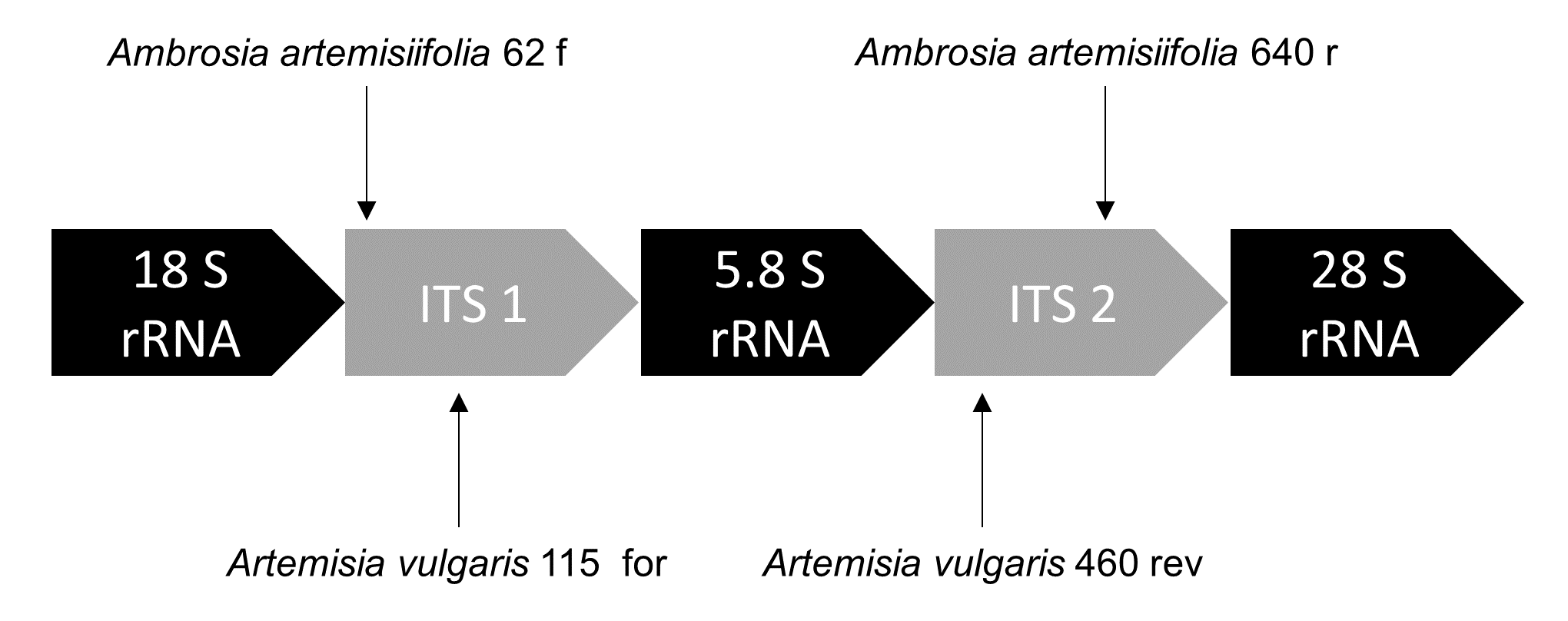

Supplement: Supplementary file 2 — Supplementary material 2 (PNG 34 kb) [file 10453_2017_9485_MOESM2_ESM.png]

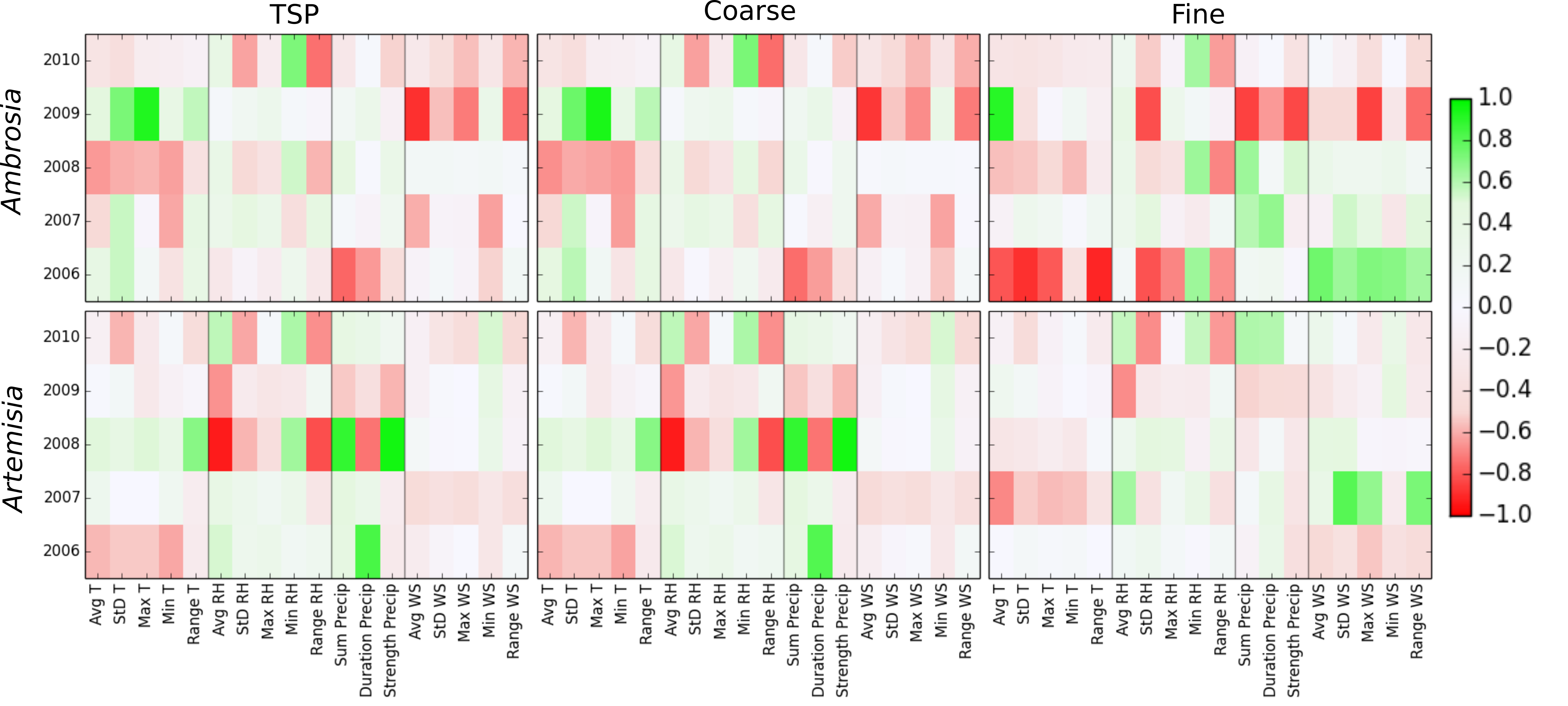

Supplement: Supplementary file 5 — Supplementary material 5 (PNG 985 kb) [file 10453_2017_9485_MOESM5_ESM.png]
